# Transcriptional Profiling of Rat Prefrontal Cortex after Acute Inescapable Footshock Stress

**DOI:** 10.3390/genes14030740

**Published:** 2023-03-17

**Authors:** Paolo Martini, Jessica Mingardi, Giulia Carini, Stefania Mattevi, Elona Ndoj, Luca La Via, Chiara Magri, Massimo Gennarelli, Isabella Russo, Maurizio Popoli, Laura Musazzi, Alessandro Barbon

**Affiliations:** 1 Department of Molecular and Translational Medicine, University of Brescia, 25123 Brescia, Italy; 2 Department of Medicine and Surgery, University of Milano-Bicocca, 20900 Monza, Italy; 3 Genetics Unit, IRCCS Istituto Centro San Giovanni di Dio Fatebenefratelli, 25123 Brescia, Italy; 4 Department of Pharmaceutical Sciences, University of Milan, 20133 Milan, Italy

**Keywords:** acute stress, footshock, transcriptional profiling, microarray, transcriptional factors, prefrontal cortex, stress-related disorders

## Abstract

Stress is a primary risk factor for psychiatric disorders such as Major Depressive Disorder (MDD) and Post Traumatic Stress Disorder (PTSD). The response to stress involves the regulation of transcriptional programs, which is supposed to play a role in coping with stress. To evaluate transcriptional processes implemented after exposure to unavoidable traumatic stress, we applied microarray expression analysis to the PFC of rats exposed to acute footshock (FS) stress that were sacrificed immediately after the 40 min session or 2 h or 24 h after. While no substantial changes were observed at the single gene level immediately after the stress session, gene set enrichment analysis showed alterations in neuronal pathways associated with glia development, glia–neuron networking, and synaptic function. Furthermore, we found alterations in the expression of gene sets regulated by specific transcription factors that could represent master regulators of the acute stress response. Of note, these pathways and transcriptional programs are activated during the early stress response (immediately after FS) and are already turned off after 2 h—while at 24 h, the transcriptional profile is largely unaffected. Overall, our analysis provided a transcriptional landscape of the early changes triggered by acute unavoidable FS stress in the PFC of rats, suggesting that the transcriptional wave is fast and mild, but probably enough to activate a cellular response to acute stress.

## 1. Introduction

Stress is a physiological response to any condition that perturbs the homeostasis of a living organism. When rapidly activated and then shut off, the stress response is proadaptive, but it may become maladaptive when the stressful stimulus is repeated or overwhelming or in subjects with a genetic background of vulnerability [1]. Accordingly, stress is considered a primary risk factor for many psychiatric disorders, including Major Depressive Disorder (MDD) and Post Traumatic Stress Disorder (PTSD) [1]. The prefrontal cortex (PFC)—a region involved in working memory, decision-making, social interaction, and emotional processing—is a main target of stress [2,3,4,5]. Increasing evidence has consistently shown that the fast response to stress involves increased attention, vigilance, and improved PFC-mediated cognitive performance [6]. In previous studies, we have deeply characterized the functional and morphological changes induced in the PFC of rats by acute inescapable footshock (FS)—a widely used animal model of PTSD [7,8,9]. We demonstrated that FS induced a rapid and long-lasting enhancement of glutamate release in the PFC, already measurable immediately after stress exposure and for at least up to 24 h after [3,4,5]. FS also induced time-dependent modulation of both AMPA (α-amino-3-hydroxy-5-methyl-4-isoxazolepropionic acid) and NMDA (N-methyl-D-aspartate) receptor subunit expression and phosphorylation, suggesting an early and transient enhancement of AMPA receptor-mediated currents followed by a transient activation of NMDA receptors [10]. Interestingly, these functional alterations of glutamatergic transmission were accompanied by dendritic atrophy and retraction, observed as early as 24 h after FS and sustained for at least 14 days [11]. Using positron emission tomography (PET), we also found a rapid increase in synaptic energy metabolism in the PFC and rapid and sustained alterations in working memory performance [12].

However, the detailed molecular mechanisms underlying these changes have not yet been fully elucidated and, to the best of our knowledge, the genome-wide expression profile in the PFC after acute traumatic stress in rats has never been investigated before.

In this work, we performed microarray global transcriptome profiling in the PFC of FS-stressed rats to identify time-dependent transcriptional programs and associated pathways underlying FS-dependent molecular alterations that could take part in the response to acute stress. Genome-wide expression profiles of the PFC were obtained immediately after the 40 min FS session, as well as 2 and 24 h after the initiation of stress, to allow the monitoring of longitudinal changes in expression induced by acute traumatic stress. 

While no substantial statistically significant changes were observed at the single gene level immediately after stress exposure, gene set enrichment analysis (GSEA) showed alterations in neuronal pathways associated with neuronal morphology and synaptic function. Furthermore, we found alterations in the expression of gene sets regulated by specific transcription factors (TFs), which could represent master regulators of the acute stress response. Finally, we observed that the molecular mechanisms activated to face acute stress are switched off after 2 h, while at 24 h, the transcriptional profile is mainly unaffected.

## 2. Materials and Methods

### 2.1. Animals

All experimental procedures involving animals were performed in accordance with the European Community Council Directive 2010/63/UE and approved by Italian legislation on animal experimentation (Decreto Legislativo 26/2014, authorization N 521/2015-PR). Experiments were performed on adult male Sprague–Dawley rats (275–300 g). Rats were housed two per cage and maintained in a 12/12 h light/dark schedule (lights on at 7:00 am), in a temperature-controlled facility with free access to food and water. The experiments were performed during the light phase (between 9:00 and 12:00 am) at least one week after arrival from the supplier (Charles River, Wilmington, MA, USA). 

### 2.2. Footshock (FS) Stress Procedure

The footshock (FS)-stress protocol was performed essentially as previously reported (40 min FS stress: 0.8 mA, 20 min total of actual shock with random inter-shock length between 2–8 s) [4,10,13]. Control rats were left undisturbed in their home cages. Rats were killed by decapitation at different time points (the number of animals involved in each experiment is reported in the figure legends): immediately after the stress session (40 min), and 2 or 24 h after the initiation of stress. The 2 and 24 h groups were put back in their home cages after the 40 min stress session until sacrifice. At sacrifice, the PFC was quickly dissected on ice and alternatively assigned to RNA extraction or protein purification.

### 2.3. RNA Extraction and Purification

Total RNA from the rat PFC was isolated by single-step extraction using TRIzol Reagent (Life Technologies, Milan, Italy), according to the manufacturer’s instructions. Subsequent RNA clean-up was performed using the RNeasy mini kit (QIAGEN, Milan, Italy) to obtain high quality RNA.

RNA quantification and quality controls were carried out using both spectrophotometric analysis (Nanodrop 2000, Nanodrop Technologies, Wilmington, DE, USA) and AGILENT Bioanalyzer 2100 lab-on-a-chip technology (AGILENT Technologies, Santa Clara, CA, USA) [14]. The purity of each sample was determined by assessing the A260:280 ratio, with acceptable values ranging from 1.8 to 2.2.

### 2.4. Microarray Procedures

For microarray, 250 ng of the total RNA of each sample was processed using Ambion WT Expression Kits for amplification and using the Affymetrix Whole Transcript (WT) Sense Target Labeling Assay Kit (Life Technologies) to prepare an adequate amount of the labeled target for hybridization [15]. Briefly, total RNA was reverse-transcribed into double-stranded cDNA using random hexamers (thus avoiding the 3′-bias introduced using oligo-dT primers) tagged with a T7 promoter sequence. Then, the double-stranded cDNA was amplified by T7 RNA polymerase in an in-vitro transcription reaction to produce antisense cRNA. In the second cycle of cDNA synthesis with random hexamers, cRNA was reverse-transcribed into single-stranded DNA in the sense orientation. Then, 5.5 µg ssDNA was fragmented, terminally labeled with biotin, hybridized, and processed using Affymetrix GeneChip Rat Gene 1.0 ST Arrays. After washing and staining with fluorescent streptavidin using the Affymetrix GeneChip Fluidics Station 450, chips were scanned with the GeneChip Scanner 3000 7G. Fluorescent signals were acquired by Affymetrix GeneChip Command Console (AGCC) software. Microarray analysis was performed on the samples obtained immediately after the 40 min FS session (*N* = 8 FS vs. *N* = 8 controls), as well as 2 h (*N* = 6 FS vs. *N* = 6 controls) and 24 h (*N* = 5 FS vs. *N* = 5 controls) after the beginning of the stress session.

### 2.5. Microarray Expression Analysis

Gene expression was read and quantified from CEL files using the read.celfiles function from the oligo R package (version 1.58.0) [16]. Data were normalized using the rma function from the oligo R package. Probe sets were annotated using the ragene10sttranscriptcluster.db R package (version 8.8.0). We kept only the probe sets with normalized expressions greater than 4. Principal component analysis (PCA) was performed using the sva function from the R base package. Differential expression analysis was performed with the limma R package (version 3.50.3), using the empirical Bayes moderated t-statistics test (eBayes function) [17]. Adjusted *p*-values were computed using the Benjamini–Hochberg method. 

Gene set analysis was performed with the ClusterProfiler R package (version 4.2.2) [18]. Gene Ontology (GO) gene sets were defined using the org.Rn.eg.db R annotation package. KEGG pathway gene sets were derived from the graphite R package (version 1.40.0) [19,20]. Transcription Factor (TF) gene sets for rats were retrieved from the msigdbr R package (version 7.5.1), which uses MSigDB regulatory gene sets (C3 category) [21,22].

We used gseGO for GSEA with the GO and GSEA function for KEGG and transcription factor (TF) target analysis. Both GSEA functions implement the GSEA method described in [22]. For both functions, additional parameters were set as follows: *p*AdjustMethod = “BH” (Benjamini–Hochberg method), *p*valueCutoff = 0.1 (adjusted *p*-value significance threshold), minGSSize = 10, and maxGSSize = 500 (min and max dimension of gene set analyzed).

TF activity was computed using the Gene set variation analysis (gsva) method from the GSVA R package (version 1.42.0) [23]. This method allows the computation of a single sample gene set enrichment score for the TF targets gene set. The enrichment score was used as a proxy of TF activity. A heatmap was created using the pheatmap R package, using gsva scores transformed in Z-score.

### 2.6. Western Blotting: Tissue Processing and Image Analysis

PFC tissue was homogenized 1:10 (*w*/*v*) by a loose-fitting Potter in homogenization buffer (0.28 M sucrose buffered at pH 7.4 with Tris, containing phosphatase inhibitors (Thermo-Fisher Scientific, Milano, Italy) and 2 mL/ml of protease inhibitor cocktail (Merck-Millipore, Milano, Italy)). Protein concentrations were evaluated by Bradford or BCA assays (Merck-Millipore and Thermo Fisher Scientific, Milano, Italy, respectively) and 10–30 micrograms were loaded onto acrylamide SDS-PAGE gels. Western blotting was performed as previously described [10,24]. Specific primary antibodies used were: AKT (1:1000, Cell Signaling cod. 4691); pAKT (1:1000 Cell Signaling cod. 4056); CaM kinase II (1:1000 Chemicon cod: AB3111); pCaM kinase II (1:1000 Thermo Scientific cod: PA14614); CREB (1:1000 Cell Signaling cod: 91975); pCREB (1:1000, Cell Signaling cod: 91985); ERK (1:1000, Cell Signaling cod: 46955); pERK (1:1000, Cell Signaling cod: 43705); GR (1:1000 Santa Cruz cod. sc-1004); pGR (1:1000 Cell Signaling cod. 4161); MR (1:1000 Santa Cruz cod: sc-114112); and mGluR (1:1000, Abcam cod. ab15672). Antibodies against β-actin (1:40,000, Merck-Millipore, cod. Mab374) were used as an internal control.

Incubation with primary antibodies was carried out overnight at 4 °C. Membranes were washed five times with TBS-Tween 20 0.2% and incubated for 1 h at room temperature with AP-conjugated secondary antibodies (Promega, Milan, Italy). Immunolabeled proteins were detected by incubation with Supersignal West Pico Chemiluminescent Substrate (Pierce, Rockford, IL, USA) or CDPStar (Roche Applied Science, Monza, Italy) detection reagents. The intensity of immunoreactive bands was analyzed with Image-Pro Plus. Data are presented as optical density ratios of the investigated protein band, normalized by β-actin bands in the same line, and are expressed as a percentage of controls.

### 2.7. Statistical Analysis

All the statistical analyses were performed using the R environment for statistical computing and graphics (version 4.1.3), unless otherwise stated. For differential expression analysis, we used the empirical Bayes moderated t-statistics test (eBayes function) with *p*-values adjusted using the Benjamini–Hochberg method, implemented in limma [17]. For gene set/pathway analysis, we used GSEA [22], using log-fold-changes with *p*-values adjusted using the Benjamini–Hochberg method, implemented in the clusterProfiler R package. 

For Western blot experiments, statistical data analysis was carried out using GraphPad Prism 9 (GraphPad Software Inc., San Diego, CA, USA). Results are presented as means ± standard error of the mean (SEM). Welch’s *t*-test was used to compare the protein expression levels of FS vs. control animals.

## 3. Results

### 3.1. Gene Expression Analysis in the PFC Immediately after Footshock Stress

The PFC transcriptomes of rats subjected to FS stress were compared to controls immediately after the 40 min FS session. PCAs using both the most variable genes and the whole transcriptome did not clearly separate controls from FS rats (Supplementary Figure S1). Differential expression analysis between stressed and control rats confirmed this observation, as we found no significant differentially expressed genes (DEGs; adjusted *p*-value ≤ 0.05; summary statistics of the transcriptomic analyses are reported in Supplementary Table S1). Looking at the most up-regulated genes (i.e., those genes more highly expressed in the FS PFC compared to controls), we found a number of neuronal genes that encode for proteins that localize at synapses such as Drd1 and Drd2 (Dopamine receptor D1 and D2-log fold-change FS vs. controls of 1.13 and 1.31; *p*-value 0.05 and 0.051; adjusted *p*-value 0.54 and 0.54, respectively). Among the down-regulated genes, we observed that Grm2 (glutamate metabotropic receptor 2) was the most down-regulated (log fold-change FS vs. controls of −0.92; *p*-value 0.0169; adjusted *p*-value 0.51).

### 3.2. Gene Set Enrichment Analysis in the PFC of Rats Immediately after Footshock Stress

We performed a Gene Set Enrichment Analysis (GSEA) to look for gene sets or biological pathways with small yet coordinated trends of up- or down-regulation without specifying a fixed threshold. As described in the original method [22], GSEA does not need to specify a list of differentially expressed genes, but works on the whole list of analyzed genes.

**Figure 1:**
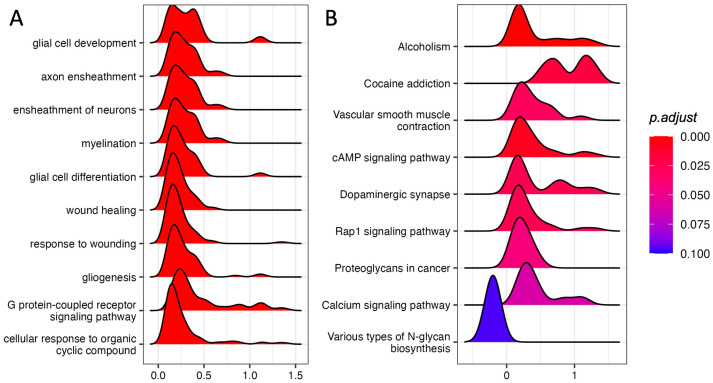
GSEA in the PFC of rats immediately after acute FS stress. Density distribution of log fold-changes for the core genes belonging to the gene sets reported. On the x-axis, the log fold-change is reported. (**A**) Top 10 enriched GO Biological Process terms. (**B**) KEGG-enriched pathways with adjusted *p*-value ≤ 0.1. *N* = 8 FS vs. *N* = 8 CTR.

We performed GSEA using the GO Biological Process annotation. Among the top 10 most significant pathways (adjust *p*-value ≤ 0.1) we found “glia cell development”, “axon ensheathment”, “ensheathment of neurons”, and “glial cell differentiation”, suggesting that glia–neuron responses could be involved in the response to acute stress (Figure 1A; Supplementary Table S2).

We also performed GSEA using KEGG pathways, finding nine significant pathways with an adjusted *p*-value ≤ 0.1 (Figure 1B; Supplementary Table S3). Strikingly, five out of eight up-regulated pathways were involved in neuronal activity. 

**Figure 2:**
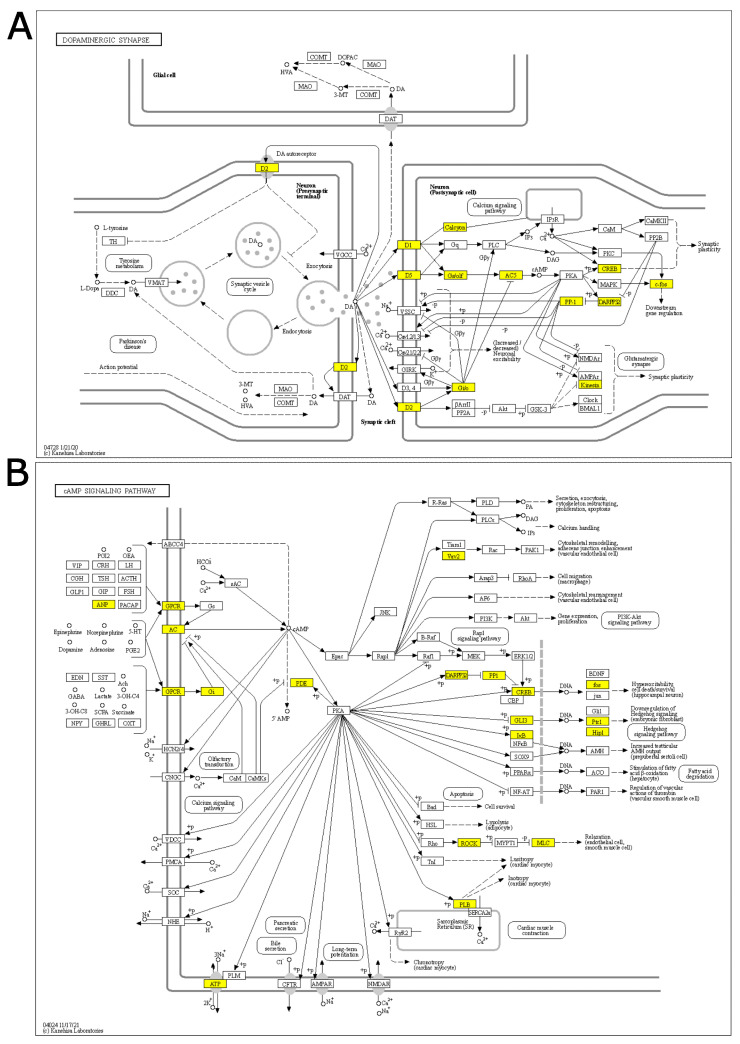
KEGG core enrichment signal chains in the PFC of rats immediately after acute FS stress. KEGG pathway of the (**A**) Dopaminergic synapse pathway and (**B**) cAMP signaling pathway, derived from the 40 min analysis. Yellow-highlighted genes belong to the core enrichment of the gene set (i.e., leading edge genes that drive enrichment). *N* = 8 FS vs. *N* = 8 CTR.

“Dopaminergic synapse”, “Alcoholism”, and “Cocaine addiction” are pathways involving dopaminergic signaling and are ruled by the genes Drd1 and Drd2, which also show the highest log fold-changes (1.13 and 1.31, respectively; Figure 2A, Supplementary Figure S2) and belong to the core enrichment (i.e., among the most up-regulated) of the three pathways. Dopaminergic activation may imply the involvement of the cAMP signaling pathway, which is also enriched in our GSEA analysis. In the core enrichment of “Dopaminergic synapse”, we also found the two transcription factors c-fos (encoded by Fos, Fos proto-oncogene, AP-1 transcription factor subunit) and CREB (encoded by Creb1: cAMP responsive element binding protein 1), as well as Kif5b and Ppp1r1b (Kinesin and PP-1 on the KEGG map, respectively; Figure 2A), that are known to play crucial roles in the regulation of synaptic activity and plasticity [25,26]. Other up-regulated KEGG pathways included calcium signaling and signal transduction cascades. The only down-regulated (and the least significant) pathway was related to N-glycan biosynthesis.

### 3.3. Gene Expression Analysis and Gene Set Enrichment Analysis (GSEA) in the PFC of Rats 2 and 24 h after Acute Footshock Stress

To evaluate the transcriptional changes induced by acute FS in the PFC over time, we examined the PFC transcriptome of FS-stressed animals 2 and 24 h after the beginning of the stress session. Results for differential expression analysis at 2 h and 24 h are reported in Supplementary Table S4 and S5, respectively. Overall, we found seven differentially expressed genes at 2 h and none at 24 h (adjusted *p*-value ≤ 0.05). Of note, Sgk1—a gene that has been previously reported to be up-regulated after acute stress—is among the DEGs observed at 2 h. GSEA revealed no activated processes 2 h after stress. On the contrary, we observed that many of the processes activated immediately after the stress session were significantly down-regulated 2 h later, including GO Biological Processes related to neurotransmitter trafficking such as “response to monoamine”, “response to dopamine”, and “response to catecholamine”, as well as KEGG pathways for “Cocaine addiction”, “Calcium signaling pathway”, and “Alcoholism” (Supplementary Figure S3; Supplementary Tables S6 and S7). Of note, the core-enrichment genes of the “Cocaine addiction” pathway largely overlapped with those found immediately after stress (Supplementary Figure S2A). Similarly, 2 h after stress, the expression of *Grm2—*the most down-regulated gene immediately after stress—was completely restored, going from a log fold-change FS vs. control of −0.92 immediately after stress (*p*-value 0.01688411, adjusted *p*-value 0.5152705) to 0.16 2 h later (*p*-value 0.56, adjusted *p*-value 0.96).

At 24 h after FS, we found no sign of the transcriptional perturbation seen at 40 min. Pathway analysis mainly evidenced pathways related to RNA biosynthesis and inflammation (Supplementary Figure S4; Supplementary Tables S8 and S9). 

### 3.4. Transcriptional Factor Gene Target Analysis

Since most of the variations were observed immediately after the acute FS stress session, we deepened our analysis at this time point. We observed that several pathways activated immediately after acute FS stress pointed to the up-regulation of downstream TFs, including CREB and cFos. Therefore, we investigated if the targets of TFs were activated or repressed in response to FS.

**Figure 3:**
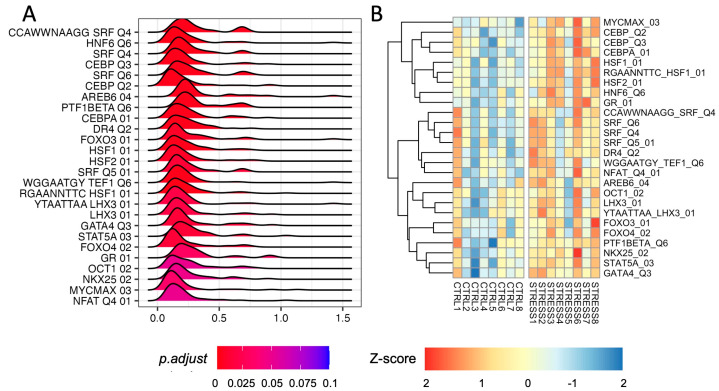
Transcription Factor analysis in the PFC of rats immediately after acute FS stress. (**A**) Density distribution of log2 fold-changes of the core enriched genes belonging to the TF target gene sets (adjusted *p*-value ≤ 0.1). On the x-axis, the log2 fold-change is reported. (**B**) TF activity scores computed using the “gsva” method from the GSVA R package for TFs, with significant enrichment of the TF target gene set.

We performed GSEA using the regulatory set from the molecular signature database (MSigDB) [21,22]. We found a significant upregulation of several gene targets of TFs (adjusted *p*-value ≤ 0.1; Figure 3A; Supplementary Table S10). We found an enrichment of Serum Response Factor (SRF) target gene sets (adjusted *p*-values 0.0055)—a TF capable of modulating Egr1/2 and cFos expression as well as that of the Glucocorticoid Receptor (GR; adjusted *p*-values 0.031). We also found an enrichment for HSF1 and HSF2 target genes (adjusted *p*-value 0.0055, 0.0098 respectively). However, we did not find any CREB- or c-Fos (AP-1) target enrichment. Our analysis also highlighted the enrichment of CEBP targets (adjusted *p*-value 0.0066) that—to the best of our knowledge—has never been associated with acute stress in rats. We did not detect any TF target gene set that was significantly down-regulated.

To evaluate the level of activation of TFs in each sample, we inferred TF activity by computing the sample-wise activity scores of all significant TFs. As shown in Figure 3B, an overall increased activity of GR, SRF, HSF1/2, and CEBP TFs was found in stressed animals.

### 3.5. Protein Expression Analysis of Stress Response Key Effectors in the PFC of Rats Immediately after Acute FS Stress

To evaluate how the transcriptional wave might be implemented into a protein response, we selected key regulators identified by pathway and TF analyses and measured their protein expression levels in the PFC of rats immediately after FS stress by Western blotting.

**Figure 4:**
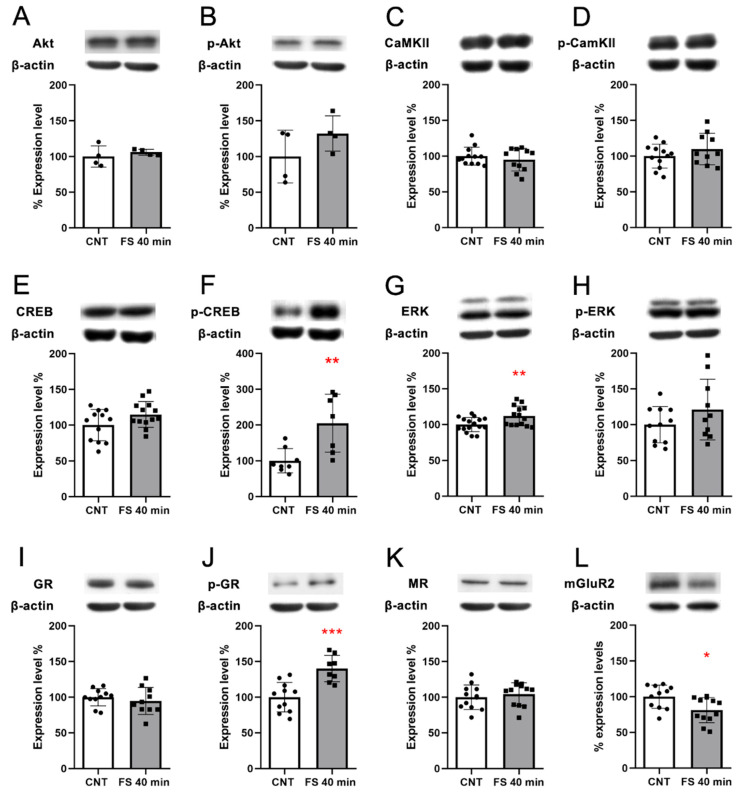
Protein expression analysis of key effectors of the stress response in the PFC of rats immediately after acute FS stress. Akt protein expression (**A**) and phosphorylation (**B**); CaM kinase II (CaMKII) protein expression (**C**) and phosphorylation (**D**); CREB protein expression (**E**) and phosphorylation (**F**); ERK protein expression (**G**) and phosphorylation (**H**); GR protein expression (**I**) and phosphorylation (**J**); MR protein expression (**K**); mGluR2 protein expression (**L**). Insets: representative WB bands. Data are represented as percentage of controls as means ± SEM. Welch’s t test was used for statistical analysis (*n* = 10; AKT and pAKT *n* = 4). * *p* < 0.05; ** *p* < 0.01; *** *p* < 0.001.

As key terminal regulators of the activated pathways “Dopaminergic Synapse”, “cAMP signaling pathway”, and “Calcium signaling pathway”, we selected calcium calmodulin (CaM) kinase 2a and Akt and measured both their total protein expression levels and their activation by phosphorylation. We observed no significant changes (Figure 4A–D).

Moreover, we evaluated if the activation of the cAMP signaling pathway triggered an increase in CREB protein levels and its activation by phosphorylation. Although we did not observe any increase in CREB protein levels, we observed a significant increase in CREB phosphorylation levels in FS animals compared to controls (Welch’s *t*-test *p* < 0.01 Figure 4E,F, respectively), indicating that FS induced a rapid activation of CREB. Other kinases that are activated downstream of the cAMP signaling pathway are the ERKs, which are also responsible for the phosphorylation of CREB [27]. We observed a significant increase in ERK protein expression levels (Welch’s *t*-test *p* < 0.05) and a non-significant trend in increased ERKs phosphorylation (Figure 4G,H, respectively).

TF target analysis showed several active TFs in FS-stressed animals immediately after stress; therefore, we tested the protein expression of the glucocorticoid receptor (GR) and mineralocorticoid receptor (MR), which are directly activated by corticosterone—the main stress hormone [28]. We observed no changes in GR (Figure 4I) and MR (Figure 4K) protein levels, while pGR significantly increased after FS (Welch’s *t*-test *p* < 0.001; Figure 4J)—suggesting its activation by phosphorylation, which is in line with the TF target gene set analysis.

In the transcriptome analysis, we observed the downregulation of *Grm2* transcription. This reduction was also confirmed at the protein expression level (Welch’s *t*-test *p* < 0.05; Figure 4L).

## 4. Discussion

In the present study, we report a comprehensive analysis of the PFC transcriptomic profile in rats subjected to acute inescapable stress. We used the standardized FS stress model, which we have already dissected at both the functional and morphological level [3,4,5,10,11]. However, to the best of our knowledge, a global transcriptomic analysis in the PFC of this model has never been performed before. To shape the transcriptional wave following stress exposure, we looked at transcriptional changes immediately after the 40 min stress session, as well as 2 and 24 h after the initiation of stress [11].

Our results showed that neither immediately after FS stress, nor 2 or 24 h after, could substantial changes at the single-gene level be detected. This suggests that—in face of rapid and long-lasting functional, structural, and protein changes induced by acute FS in the PFC of rats [7,9]—transcriptional changes seem to be mild. 

Previous studies investigating the time-dependent transcriptional effects of acute stress have mainly been conducted in mice (although a few reports on rats are also available) and have essentially focused on the hippocampus [29,30,31,32,33]. Other brain areas have been investigated—including the amygdala, nucleus accumbens, and locus coeruleus—but the number of reports is small [34,35,36,37,38]. 

Although limited due to the use of microarrays instead of next-generation sequencing methods, to the best of our knowledge this is the first study describing the transcriptional signature of acute stress in the PFC. Of note, the previous literature on acute stress presents few points of convergence, basically reporting transient increases in a limited number of genes—mainly immediate–early genes [39,40,41,42,43,44]. 

Interestingly, a recent bioinformatic study analyzed the transcriptional profile associated with different stress conditions in mice and reported high variability in the pattern of gene expression after FS exposure and, remarkably, a different set of DEGs was obtained for each region, with a limited intersection between different studies [45]. This suggests that the transcriptional signature of stress is not only strictly dependent on the brain area analyzed, but also on the specific stress protocol applied and the sex, age, species, and strain of the animals used [1].

Future studies are required to unveil a more complex picture of the transcriptional response of the PFC to acute stress. For example, in the present study, we analyzed the whole PFC, without dissecting functional subregions (e.g., infralimbic or prelimbic PFC) or considering single-cell transcriptomic profiling. In this context, in a recent study, a large portion of the active transcriptional response to acute stress in the hippocampus was found to be driven by non-neuronal cell types—particularly vascular cells and astrocytes [46]. In our model, performing GSE Analysis—thus looking for gene sets or biological pathways with small yet coordinated trends of up- or down-regulation without specifying a fixed threshold—allowed the identification of pathways modulated by FS in the PFC of rats.

Immediately after stress, we found upregulation of GO Biological Process terms such as “glia cell development”, “axon ensheathment”, “ensheathment of neurons”, and “glial cell differentiation”, which indicates the activation of gene sets that promote glial adaptation and glial/neuronal remodeling. Alterations in glial function have been implicated in mental disorders [47,48] as well as in the adaptive response to acute stress [49,50,51]. Our data are in line with the literature, indicating a role of acute stress in reshaping neuron–glia networking in the PFC. Furthermore, by analyzing KEGG pathways, we found eight up-regulated pathways and one down-regulated pathway. Strikingly, five out of eight up-regulated pathways (“Dopaminergic synapse”, “Alcoholism”, “Cocaine addiction”, “cAMP signaling pathway”, and “Calcium signaling pathway”) have a connection with synapse plasticity, memory, and neuronal responses to stress [52,53,54,55]. The remaining three pathways are significant mainly for their signal transduction cascades. The only down-regulated pathway is related to N-glycan biosynthesis, which has recently been associated with brain physiology and disorders [56].

The above mentioned pathways involve a high number of protein effectors, including CaM kinase II and Akt. Even if these two genes are not part of the core enrichment—given their centrality in these pathways—we analyzed their protein and phosphorylation levels, not finding any significant change immediately after stress. This may suggest that the CaM kinase 2a and Akt pathways are not directly involved in the early response to FS stress.

To further understand the transcriptional response immediately after acute FS stress, we investigated the identified pathways, focusing on the genes in the core enrichment. We observed the activation of the Drd1 and Drd2 genes, coding for dopamine receptor 1 and 2 of the dopaminergic synapse, which are also key elements of the cocaine addiction- and alcoholism-related pathways. In the dopaminergic synapse, this chain of activation links to synapse plasticity and to the cAMP signaling pathway, which were also enriched in our GSEA analysis. Two transcription factors appear to be the final effectors of the “Dopaminergic synapse” and related pathways: c-fos (encoded by Fos, the Fos proto-oncogene and AP-1 transcription factor subunit) and CREB (encoded by Creb1: cAMP responsive element binding protein 1); both genes were in the core enrichment (i.e., the most up-regulated genes) of the pathways. Two other genes among those of the core enrichment of the Dopaminergic synapse were Kif5b and Ppp1r1b (Kinesin and PP-1 on the KEGG map). These two genes, along with the calcium signaling pathway, seem to play a crucial role in synaptic activity and plasticity—given Kif5b’s interaction with AMPA receptors and Ppp1r1b’s ability to inhibit both AMPA and NMDA receptors [57,58,59]. As a main effector of the cAMP signaling pathway, we analyzed CREB protein and ERKs, which are among the kinases that phosphorylate CREB [60]. We found that ERK proteins were significantly up-regulated in response to stress. Accordingly, we found that phosphorylated CREB, remarkably, increased soon after FS stress. Nevertheless, the CREB target gene set was not significant in our TF analysis, and thus CREB activity was not computed. Further analysis is necessary to explain this discrepancy.

Finally, gene expression analysis immediately after FS stress indicated a trend in decreased mGluR2 transcription levels that was confirmed at the protein level. This receptor is one of the main metabotropic glutamate receptors and has been both repeatedly implicated in the response to acute and chronic stress [61,62,63,64,65] and proposed as a putative target for antidepressants [66,67,68]. Taken together, our transcriptional analysis strengthens the hypothesis that both dopaminergic and glutamatergic synapses could be targets and mediators of the acute stress response [69,70].

Importantly, we observed that the transcriptional wave exhausted rapidly over time. In fact, we found that most of the pathways that were active immediately after stress were down-regulated 2 h later—while 24 h after FS stress, no modified pathways were detected. Our data indicate that acute stress triggered a fast, but mild transcriptional response that was resolved in a few hours. Accordingly, a recent multiomic approach evaluating the phospho-proteome, proteome, transcriptome, mirnome, and translatome of the mouse dorsal and ventral hippocampus after acute stress highlighted calcium signaling, ERK/MAPK signaling, cAMP signaling, and CREB as master regulators of the hippocampal acute stress response [46]. Intriguingly, in line with our study, all the observed molecular changes resolved efficiently within four hours after the initiation of stress.

To evaluate if the transcriptional changes were controlled by specific TFs, we inferred TF activity by focusing on those TFs whose target gene sets were significantly up-regulated. Of note, our analysis identified strong activity for TFs such as GR, SRF, HSF, and CEBP. The up-regulation of GR-regulated genes is not surprising in the context of the stress response, with GR being one of the main targets of corticosterone and GR activation that are necessary for the cellular stress response [71,72]. In line with this transcriptional data, we found that FS induced a significant up-regulation of GR phosphorylation—suggesting the activation of GR-dependent cellular pathways in response to FS stress.

Furthermore, a transcriptional program that seems particularly relevant for the FS stress response is regulated by SRF. SRF is a master regulator of immediate–early gene expression in response to external stimuli [73]. SRF has been implicated in responses to both chronic and acute stress [74,75]. Deletion of the SRF gene specifically in glutamatergic neurons has been reported to induce hyperactivity, decreased anxiety, and impair working memory. In response to restraint stress, locomotor behavior and corticosterone release are impaired in Srf −/− mutant mice, indicating the requirement of SRF activation for the physiological stress response [75]. Our data showing SRF gene-set activation suggests that it also has a role in the response to acute FS stress.

Another transcription factor that was found to be activated immediately after FS stress was CEBP. To the best of our knowledge, this is the first time that CEBP has been linked to the PFC transcriptional response after acute stress in rats. CEBP activation has also been reported in the mouse hippocampus 45 min after forced-swim stress [46]. Moreover, the loss of CEBP regulation has been shown to lead to abnormal synaptic function and cognitive disorders in mice [76], while in rats, it has been shown that CEBP activation of IGF-1 is necessary to promote neurite outgrowth and mitochondrial respiration in the brain cortex, which can protect against neurodegenerative disorders [77].

## 5. Conclusions

Overall, our work provides an overview of time-dependent transcriptional changes triggered by acute FS stress in the PFC of rats. We observed that transcriptional changes are fast, but mild, and resolve efficiently within 2 h after the initiation of stress. Moreover, we were able to identify a coordinated and consistent activation of transcriptional programs that may be involved in the response to FS. We found an involvement of dopaminergic and glutamatergic synapses as well as of cAMP signaling, which have also been found in previous works. Finally, we detected the expression of gene sets regulated by specific transcription factors that could represent master regulators of the acute stress response. Considering the transient nature of these changes, we hypothesize that they are basically part of the adaptive response to stress—although we cannot exclude that in susceptible subjects, the cascade of events activated by this transcriptional wave could lead to maladaptive consequences and increased psychopathological risk. More studies are required to address this point.

## Data Availability

The data presented in this study are openly available at GEO (www.ncbi.nlm.nih.gov/geo) under GSE224331 accession.

## Supplementary Materials

The following supporting information can be downloaded at: https://www.mdpi.com/article/10.3390/genes14030740/s1, Figure S1: Principal Component Analysis for PFC samples immediately after FS stress; Figure S2: KEGG pathway core enrichment gene inspection; Figure S3: Gene set enrichment analysis results in the PFC of rats 2 h after FS; Figure S4: Gene set enrichment analysis results in the PFC of rats 24 h after FS; Table S1: Summary of limma statistics from differential expression analysis immediately after FS (40 min); Table S2: GSEA results for GO Biological Process annotation in the PFC immediately after FS; Table S3: GSEA results for KEGG pathways in the PFC immediately after FS; Table S4: Summary of limma statistics from differential expression analysis 2 h after FS; Table S5: Summary of limma statistics from differential expression analysis 24 h after FS; Table S6: GSEA results for GO Biological Process annotation in the PFC 2 h after FS; Table S7: GSEA results for KEGG pathways in the PFC 2 h after FS; Table S8: GSEA results for GO Biological Process annotation in the PFC 24 h after FS; Table S9: GSEA results for KEGG pathways in the PFC 24 h after FS; Table S10: GSEA results for TF target gene set in the PFC immediately after FS.
