# Supplementary material for: Transcriptional Profiling of Rat Prefrontal Cortex after Acute Inescapable Footshock Stress
Source: Genes (Basel). 2023 Mar 17;14(3):740. doi: 10.3390/genes14030740 (PMC10048409; doi:10.3390/genes14030740)
Supplement: Supplementary file 1 [file genes-14-00740-s001.zip › supplementary-figures_Rev1.pdf]

Supplementary

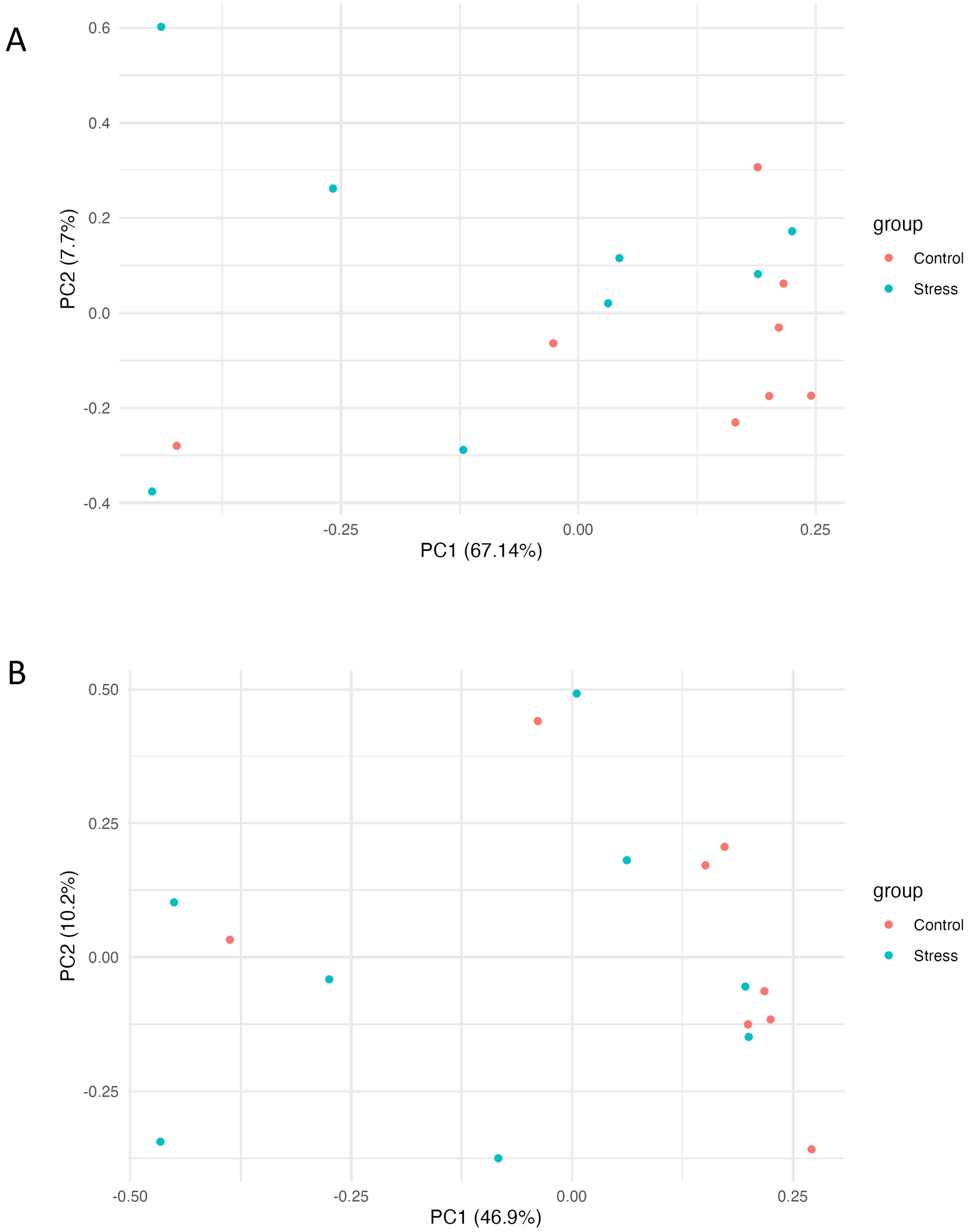

Figure S1 **Principal Component Analysis for PFC samples immediately after FS stress.** PCA plot of A) top 500 most variable genes and B) all genes respectively.

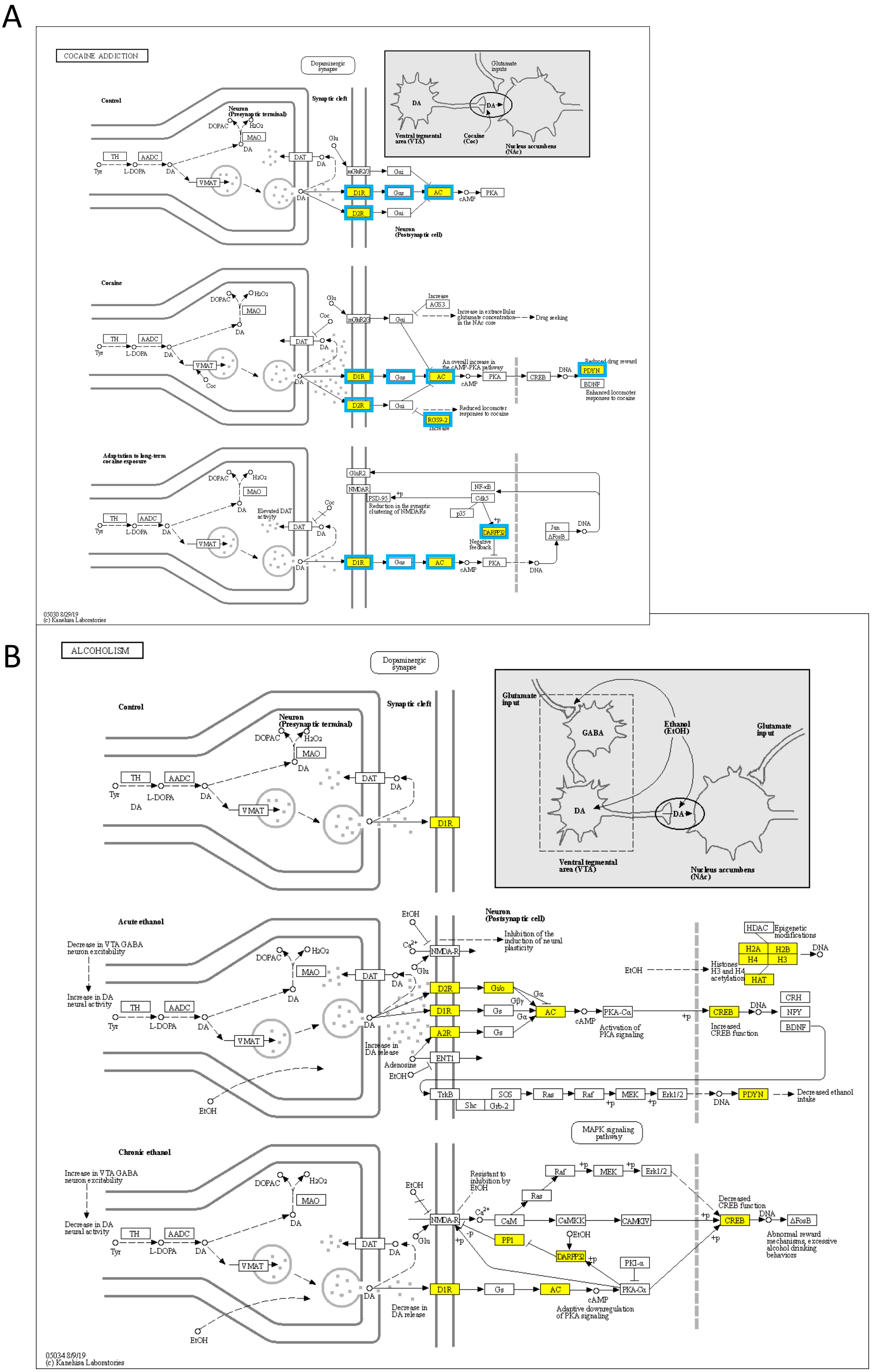

Figure S2 **KEGG pathway core enrichment gene inspection.** A) Kegg pathway of Cocaine addiction and B) Alcoholism derived from the 40 min analysis. In yellow genes that belong to the core enrichment of the pathway. Cyan borders represent repressed genes that belong to the core enrichment of the pathway and downregulated at 2h.

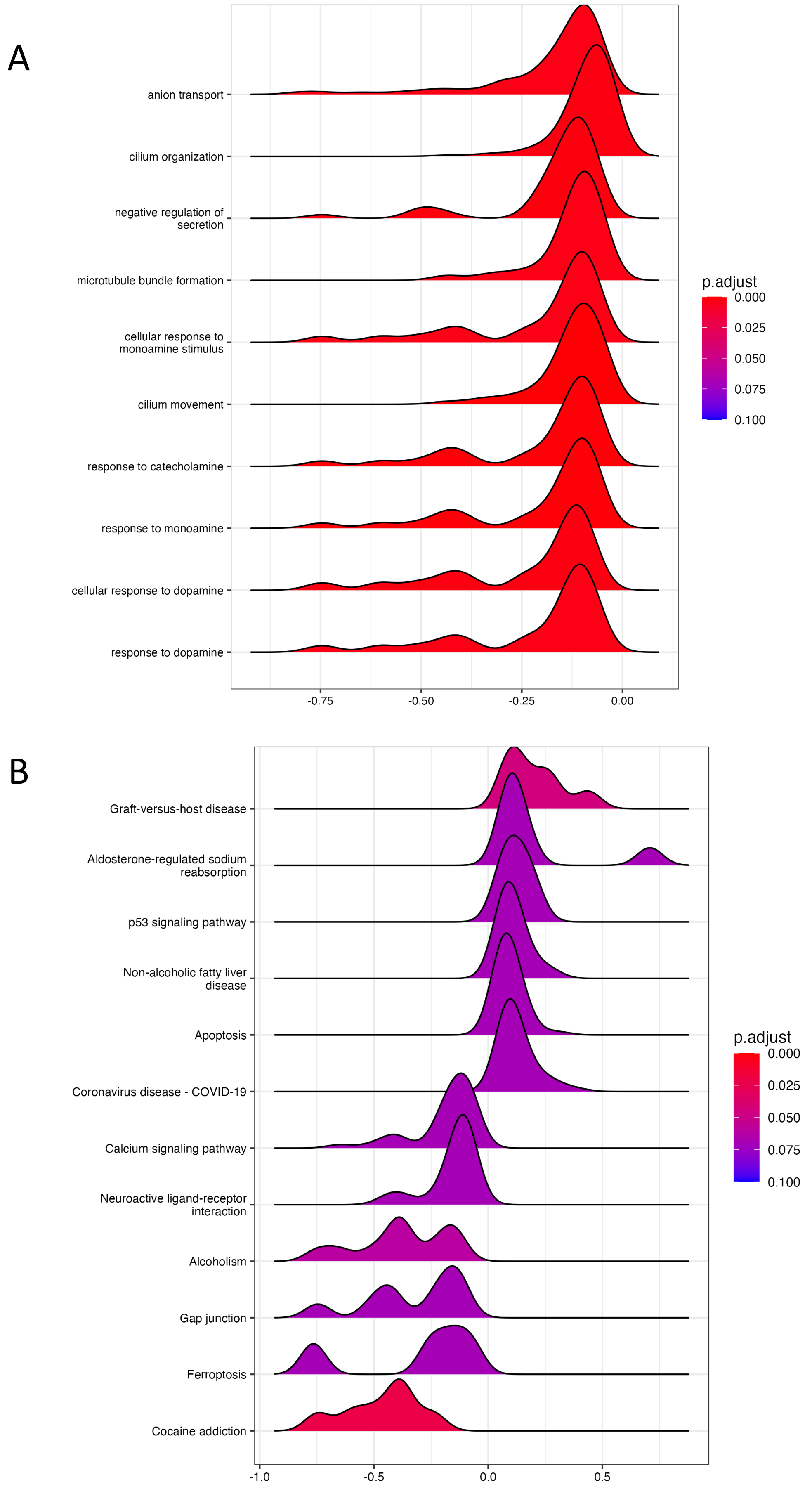

Figure S3 **GSEA in the PFC of rats 2 hours after FS stress.** Density distribution of log2 fold changes of the core enriched genes belonging to the gene sets reported. On the x axis, the log2 fold change is reported. A) Top 10 enriched GO Biological Process terms. B) KEGG enriched pathways with adjusted p-value < = 0.1. N = 6 FS vs N = 6 CTR.

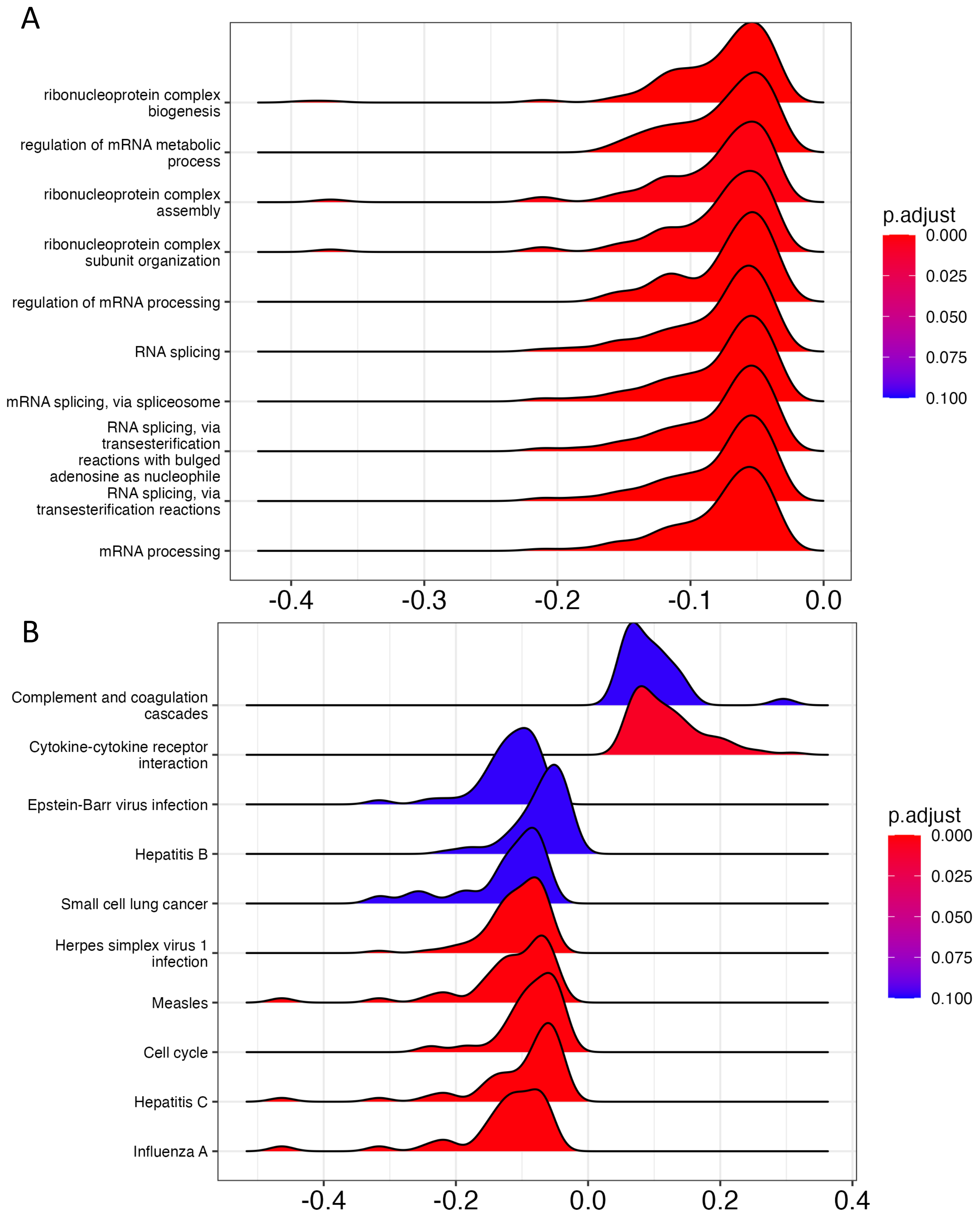

Figure S4 **GSEA in the PFC of rats 24 hour after FS stress.** Density distribution of log2 fold changes of the core enriched genes belonging to the gene sets reported. On the x axis, the log2 fold change is reported. A) Top 10 enriched GO Biological Process terms. B) KEGG enriched pathways with adjusted p-value < = 0.1. N = 5 FS vs N = 5 CTR.
